# A ‘hidden’ ^18^O-enriched reservoir in the sub-arc mantle

**DOI:** 10.1038/srep04232

**Published:** 2014-02-28

**Authors:** Chuan-Zhou Liu, Fu-Yuan Wu, Sun-Lin Chung, Qiu-Li Li, Wei-Dong Sun, Wei-Qiang Ji

**Affiliations:** 1 State Key Laboratory of Lithospheric Evolution, Institute of Geology and Geophysics, Chinese Academy of Sciences, Beijing 100029, China; 2 Department of Geosciences, National Taiwan University, Taipei 10617; 3 State Key Laboratory of Isotope Geochemistry, Guangzhou Institute of Geochemistry, Chinese Academy of Sciences, Guangzhou 510640, China

## Abstract

Plate subduction continuously transports crustal materials with high-δ^18^O values down to the mantle wedge, where mantle peridotites are expected to achieve the high-δ^18^O features. Elevated δ^18^O values relative to the upper mantle value have been reported for magmas from some subduction zones. However, peridotites with δ^18^O values significantly higher than the well-defined upper mantle values have never been observed from modern subduction zones. Here we present in-situ oxygen isotope data of olivine crystals in Sailipu mantle xenoliths from South Tibet, which have been subjected to a long history of Tethyan subduction before the India-Asia collision. Our data identify for the first time a metasomatized mantle that, interpreted as the sub-arc lithospheric mantle, shows anomalously enriched oxygen isotopes (δ^18^O = +8.03 ± 0.28 ‰). Such a high-δ^18^O mantle commonly does not contribute significantly to typical island arc basalts. However, partial melting or contamination of such a high-δ^18^O mantle is feasible to account for the high-δ^18^O signatures in arc basalts.

Much of Earth's crust returns to the mantle through the process known as subduction. A consensus has been developed that aqueous fluids and/or hydrous melts released from downgoing slabs play an important role in the genesis of convergent margin magmas^1^,^2^,^3^,^4^,^5^. Slab-released fluids/melts, on one hand, significantly decrease the temperature of the peridotite solidus and trigger partial melting of the mantle wedge^3^,^6^. On the other hand, the fluids/melts also enrich the mantle wedge in elements concentrated in altered oceanic crust and its sedimentary cover, which eventually contribute these enriched signatures to arc magmas^1^,^4^,^5^,^7^. Various chemical (i.e., both elemental and isotopic) tracers have been used to identify the components of subducted materials in arc magmas^1^,^4^,^7^. Oxygen isotopes are a powerful tool for tracing crustal materials in arc basalts and their mantle sources, due to the big contrast of oxygen isotope compositions between crustal materials and the Earth's mantle. The Earth's upper mantle has been well constrained to a limited range of δ^18^O values (5.18 ± 0.28‰; ref. 8, 9), where δ^18^O = 1000 × ((^18^O/^16^O)_sample_/(^18^O/^16^O)_V-SMOW_ − 1) and (^18^O/^16^O)_V-SMOW_ = 0.0020052 (V-SMOW being Vienna Standard Mean Ocean Water). In contrast, the oceanic crust and its sedimentary cover can have greatly different δ^18^O values as a result of low-temperature isotopic fractionations. Low-temperature seawater altered basalts have δ^18^O values of +6 ~ +12‰ (ref. 10), whereas different sediments have δ^18^O values of +12 ~ +25‰ (ref. 11, 12). Therefore, the oxygen isotope composition of a mantle-derived mineral and melt is a potentially sensitive indicator of subducted crustal materials as its protolith or in its source^13^,^14^,^15^.

Although oxygen isotope evidence for melts directly derived from subducted slabs is rare^16^, both whole-rock and olivine phenocrysts of some arc magmas have elevated δ^18^O values relative to the upper mantle^13^,^17^,^18^. This supports the involvement of slab-derived components in the sources of these arc magmas. Previous studies^14^,^19^ have also shown that silica-rich glass/melt inclusions entrained in peridotite xenoliths from island arcs have δ^18^O values (ranging from +8.8‰ to +11.3‰) significantly higher than the upper mantle, which have been interpreted as low-degree melts from refractory mantle metasomatized by slab-derived fluids/melts. A corollary is that mantle wedges could achieve the ^18^O-rich signatures from the slab-released phases. However, peridotite xenoliths from modern subduction zones have never been reported to have δ^18^O values higher than the upper mantle. It seems that the ^18^O-enriched reservoirs in mantle wedges are not represented by peridotite xenoliths trapped by arc-related magmas.

The Tibetan Plateau was formed by the northward accretion of several terranes, i.e., the Lhasa and Qiangtang blocks (Fig. 1). The Lhasa block has experienced a prolonged history of oceanic subduction (i.e., the Neo-Tethys Ocean) before the India-Asia collision at ca 55 Ma (ref. 20, 21). An active continental margin was well developed in the southern margin of the Lhasa block, represented by the Gangdese Arc. Post-collisional potassic and ultrapotassic lavas are widely distributed in the Tibetan Plateau^20^. Peridotite xenoliths selected in this study are trapped by the Sailipu ultrapotassic lavas in Lhasa Block from the southern Tibet Plateau (Fig. 1), which were erupted at ca 17 Ma (ref. 22). The Sailipu mantle xenoliths are small in size, with diameters commonly less than 2 cm. It has been suggested that they represent a relict mantle of the Asian lithosphere, which has been metasomatized by fluids/melts liberated from the subducted Tethys ocean plate and/or the Indian continent^23^. In this study, olivines from nine Sailipu peridotite xenoliths have been analyzed for oxygen isotopes by secondary ion mass spectrometry (SIMS).

## Results

The Sailipu mantle xenoliths mainly consist of olivine and orthopyroxene (Supplementary Text). All samples except SLP105 contain phlogopite, whereas clinopyroxene has been discovered in five samples. Spinel has only been found in three samples (e.g., SLP105, SLP127 and SLP154). Spinel in both SLP127 and SLP154 is interstitial among silicates, whereas it is included in orthopyroxene in SLP105. Phlogopite occasionally shows reaction textures with spinel.

**Figure 2:**
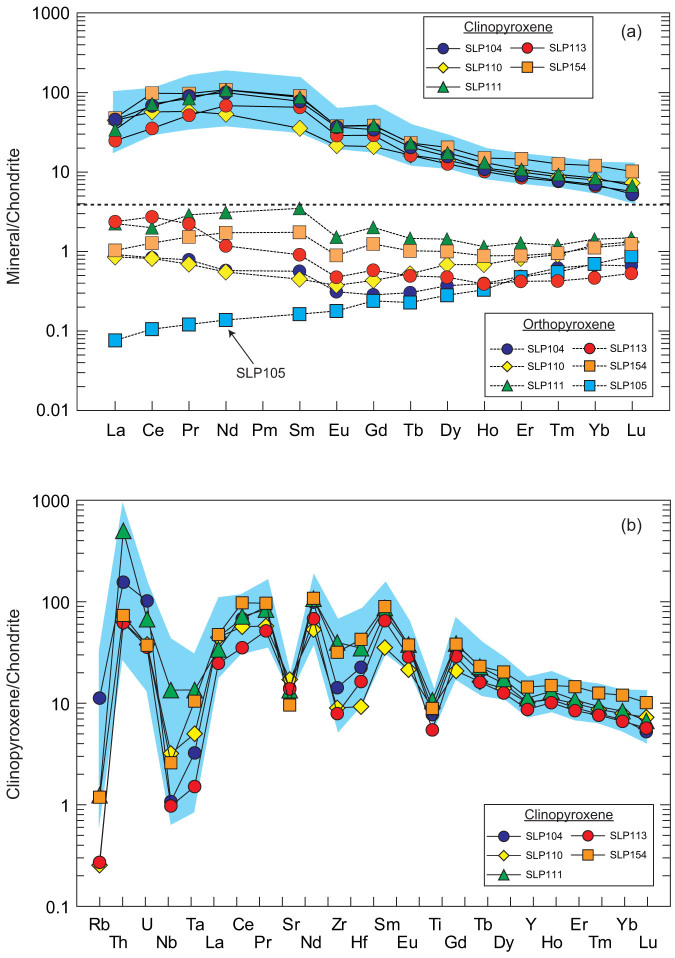
(a) Rare earth element (REE) and (b) multi-element variation patterns of pyroxenes in Sailipu mantle xenoliths. The shade areas depict the range of clinopyroxene in Sailipu mantle xenoliths reported in a previous study^23^.

Olivines in the studied mantle xenoliths have forsterite contents [Fo; = 100 × Mg/(Mg + Fe)] of 88-91 and contain 0.04–0.08 wt.% CaO, 0.12–0.16 wt.% MnO and 0.38–0.55 wt.% NiO (Supplementary Table S1). Their chemical compositions are distinctly different from olivine phenocrysts in host lavas (i.e., Fo_69–81_, 0.14–0.47 wt.% CaO, 0.24–0.44 wt.% MnO and 0.13–0.28 wt.% NiO; ref. 23). Orthopyroxenes in all samples have Mg# [ = 100 × Mg/(Mg + Fe)] values of 0.88–0.92, and contain 0.63–0.87 wt.% CaO and 0.74–4.86 wt.% Al_2_O_3_. Phlogopites in Sailipu mantle xenoliths have Mg# values of 0.86–0.9; they contain 8.31–9.49 wt.% K_2_O, 14.13–16.07 wt.% Al_2_O_3_ and 1.96–3.27 wt.% TiO_2_. Spinels in SLP127 and SLP154, have Cr# [ = Cr/(Cr+Al)] of 0.32 and 0.11, respectively; in contrast, spinel in SLP105 has a much higher Cr# of 0.73. Clinopyroxenes occurred in five samples have Mg# of 0.89–0.9; they contain 3.71–6.79% Al_2_O_3_, 0.73–0.91 wt.% Cr_2_O_3_ and 0.35–1.78% Na_2_O. They display enriched rare earth element (REE) patterns (Fig. 2a; Supplementary Table S2), similar to those reported in the previous study^23^. They are also strongly enriched in large ion lithophile elements (LILE) but depleted in high strength field elements (HFSE; Fig. 2b). Orthopyroxenes in five clinopyroxene-bearing xenoliths were also analyzed for trace elements and show variable enrichment in light rare earth elements (LREE) relative to heavy rare earth elements (HREE), whereas orthopyroxene in SLP105 displays a depleted REE pattern (Fig. 2a).

**Figure 3:**
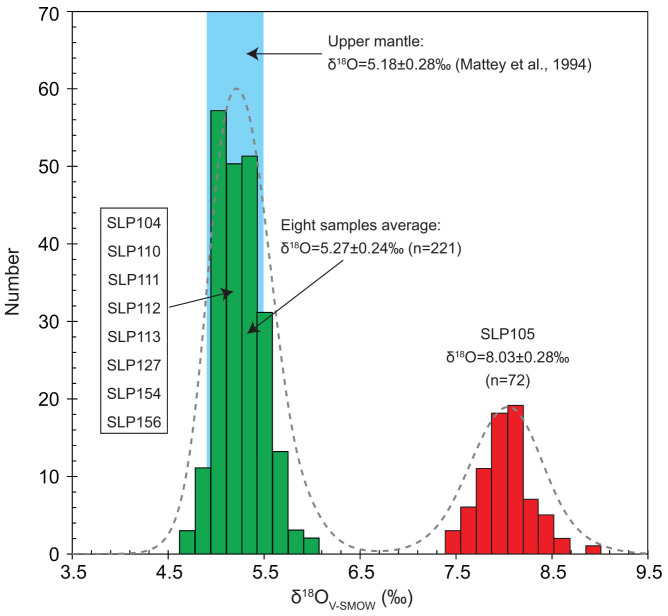
Histogram of δ^18^O values of olivines in Sailipu mantle xenoliths. The δ^18^O value of the upper mantle estimated by mantle peridotites is also shown^9^.

Olivine grains from each studied xenolith have homogeneous oxygen isotope compositions (Supplementary Table S3), as indicated by small inter- and intra-grain variations that are always less than the precision of the method (less than 0.4‰). Olivines from eight samples have δ^18^O values varying from +5.22‰ to +5.41‰ (Fig. 3), with an average of 5.27 ± 0.24‰ (n = 221). Their δ^18^O values plot within the range of the upper mantle values defined by both mantle-derived magmas and ultramafic rocks (5.18 ± 0.28‰; ref. 8, 9). In contrast, olivines from sample SLP105 with the highest Fo content also have the remarkably elevated δ^18^O values of 8.03 ± 0.28‰ (n = 72). To our knowledge, this is the highest value so far reported for mantle peridotites.

## Discussion

**Figure 4:**
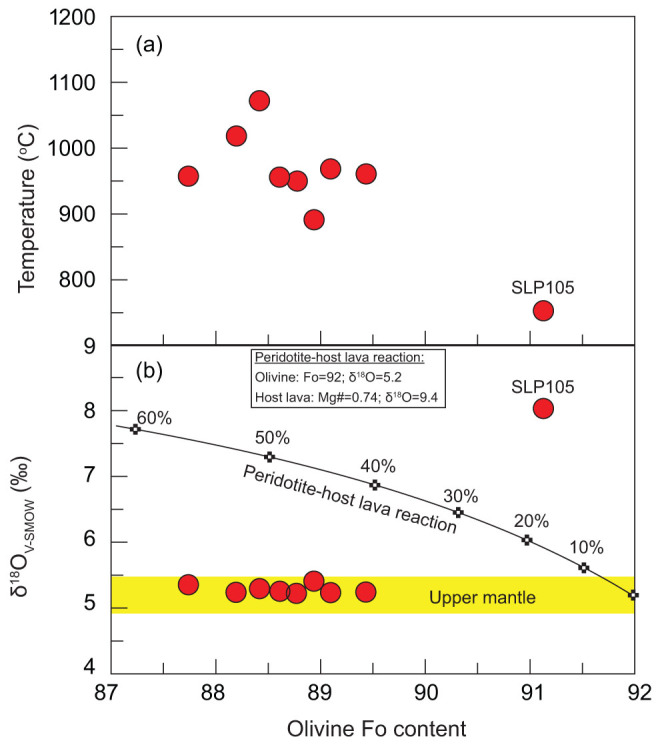
Plot of average Fo contents of olivines in Sailipu mantle xenoliths versus equilibrium temperatures (a) and oxygen isotopes of olivines (b). (a) Equilibrium temperatures were estimated using the Al-in-orthopyroxene geothermometer^24^; (b) Variation of Fo content with oxygen isotope of olivine is modeled by reaction between a mantle peridotite with a normal δ^18^O value and the host lava with an elevated δ^18^O value. The starting olivine used for modeling has a Fo content of 92 (with 50 wt.% MgO and 7.76 wt.% FeO) and δ^18^O of 5.2%; the host lava has a Mg# of 0.74 (with bulk-rock 9.51 wt.% MgO and 6 wt.% FeO) and δ^18^O of 9.4%. The data of the host lava is chosen from^22^.

Olivine in sample SLP105 could achieve the elevated δ^18^O value from the host lava, as high δ^18^O values up to 9.4‰ have been reported for the Sailipu ultrapotassic rocks^22^. Diffusion and reaction are two potential ways for host lavas to affect oxygen isotope compositions of the entrained mantle xenoliths. However, we suggest that neither process can account for the high δ^18^O value of SLP105, as explained below. Mantle xenoliths with high temperatures are more likely to achieve oxygen isotopic equilibrium with the host lavas through diffusion; thus, high-temperature mantle xenoliths are expected to achieve the oxygen isotope signatures of the host lavas. However, the equilibrium temperature of sample SLP105 estimated using the Al-in-orthopyroxene geothermometer^24^ (see Supplementary Table 1) is 753°C (Fig. 4a), which is significantly lower than temperatures estimated for the other eight samples (i.e., 891–1072°C). Therefore, the oxygen isotope compositions of sample SLP105 should be least elevated by the host lavas through diffusion, if this process really occurred. On the other hand, both olivine and orthopyroxene in mantle xenoliths would become iron-rich during reaction with ultrapotassic lavas. We note that reaction between mantle peridotites and host lavas would probably produce trends of increasing δ^18^O with decreasing Fo content in olivine (Fig. 4b) and therefore xenoliths with low-Fo olivine are expected to have elevated δ^18^O values. This is inconsistent with our observations that sample SLP105 with the highest δ^18^O have the most magnesium-rich olivine and orthopyroxene among all studied xenoliths. Therefore, reaction with host lavas could be ruled out as the cause of the elevated δ^18^O values of sample SLP105.

We suggest that olivine in SLP105 obtained its elevated δ^18^O value in the mantle through metasomatic processes by an ^18^O-rich agent. Sailipu locates on the southern margin of the Lhasa block, which experienced a prolonged history of oceanic subduction (i.e., the Neo-Tethys Ocean) before the India-Asia collision at ca 55 Ma (ref. 20, 21). Therefore, subduction is the most likely mechanism to bring such an ^18^O-rich metasomatic agent to the mantle beneath Sailipu. Trace element compositions of clinopyroxenes from five samples support that they have been highly metasomatized (Fig. 2). They show enriched REE patterns (Fig. 2a) and are also strongly enriched in large ion lithophile elements (LILE), but depleted in high strength field elements (HFSE; Fig. 2b). In combination with the presence of phlogopite in Sailipu mantle xenoliths, such trace element characteristics of clinopyroxene have been explained as a result of metasomatism by slab-derived hydrous melts^23^. However, olivines in these five clinopyroxene-bearing xenoliths and other three clinopyroxene-free xenoliths have δ^18^O values the same as the upper mantle. Therefore, a possible scenario is that slab-released hydrous melts, originally with high δ^18^O values, were in oxygen isotopic equilibrium with the ambient mantle prior to the metasomatism^25^. Orthopyroxenes from these five clinopyroxene-bearing xenoliths (i.e., SLP113) also show variably LREE-enriched patterns, which are in stark contrast to the depleted pattern displayed by orthopyroxene in sample SLP105 (Fig. 2a). This implies that trace elements of SLP105 have not been enriched by the metasomatic agent. However, the high δ^18^O value in olivine requires that it has been metasomatized by a ^18^O-rich agent. Such a decoupling between trace elements and oxygen isotopes could be reconciled if sample SLP105 was metasomatized by slab-released fluids rather than melts. It has been experimentally demonstrated^26^ that slab-released fluids could contain trace elements three orders of magnitude less than the slab-derived melts at pressures less than 4 GPa. Therefore, we suggest that sample SLP105 had been metasomatized by slab-released fluids that were not in oxygen isotopic equilibrium with the ambient mantle before metasomatism.

Sample SLP105 could achieve its high δ^18^O value in the fore-arc mantle through exchange with hydrous ^18^O-rich fluids liberated from the subducted slab at a high fluid/rock ratio^17^. A fore-arc mantle origin of SLP105 is consistent with its low temperature and the refractory composition. However, Sailipu locates at the back-arc areas of the Gangdese arc that was developed during subduction of the Neo-Tethys Ocean, which argues against that sample SLP105 was stemmed from the fore-arc mantle. Furthermore, absence of serpentine in SLP105 also does not favor its origin in a fore-arc mantle, because it has been suggested that the hydrated fore-arc mantle is mainly composed of serpentinites^27^,^28^,^29^. Peridotites in the mantle wedge just above the subducting slab could also be highly metasomatized by slab-released fluids/melts and thus have elevated δ^18^O values, which has been proposed as the source for high-δ^18^O phonolitic melts entrained in mantle olivines from the Tabar-Lihir-Tanga-Feni arc^14^. In this scenario, however, it is hard to explain how such metasomatized peridotites were incorporated into the continental mantle lithosphere beneath Sailipu. Finally, we suggest that sample SLP105 were derived from the sub-arc lithospheric mantle above the mantle wedge, which has been metasomatized by slab-derived fluids. Normally, slab-released fluids should percolate the asthenospheric wedge before they metasomatize the sub-arc lithospheric mantle. During transportation in the mantle wedge, they tend to achieve oxygen isotopic equilibrium with the ambient mantle, and thus, cannot preserve their high-δ^18^O signatures. Nevertheless, the asthenospheric wedge disappears in the flat subduction setting^30^. Therefore, the slab-released fluids could directly permeate and also transfer the high-δ^18^O signatures to the overriding lithospheric mantle. Flat subduction of the Neo-Tethys slab has been previously proposed on the basis of magmatism developed in the Gangdese arc^31^,^32^.

Our results support that some portions of the sub-arc lithospheric mantle have δ^18^O values higher than the upper mantle. Such mantle reservoirs have refractory compositions and commonly do not significantly contribute to typical island-arc basalts in normal subduction zones. This might be the reason that such an ^18^O-enriched mantle reservoir is “invisible” from oxygen isotope compositions of typical island-arc basalts^13^. In anomalous tectonic settings like arc rifting, however, this non-convecting cold mantle could melt to produce arc magmas with high δ^18^O values^17^. On the other hand, arc magmas could also achieve the ^18^O-enriched signatures through interaction (e.g., contamination) with shallow high-δ^18^O lithospheric mantle during ascending to surface^18^.

## Methods

Major elements were measured on individual minerals using a JEOL JXA-8100 electron microscope with an accelerating potential of 15 kV and sample current of 10 nA at the Institute of Geology and Geophysics, Chinese Academy of Sciences (IGGCAS). Trace elements of clinopyroxene and orthopyroxene were analyzed using a laser ablation inductively coupled mass spectrometer (LA-ICP-MS) at IGGCAS. The LA-ICP-MS system consists of a 193 nm Geolas Pro laser coupled to an Agilent 7500a ICP-MS. Isotopes were measured in peak-hopping mode. A repetition rate of 8 Hz was used during analyses for all minerals. A spot size of 80 μm was used for the analyses of clinopyroxene, whereas a spot size of 120 μm was used for orthopyroxene. The NIST 610 glass was used as an external calibration standard and isotope ^43^Ca was selected as an internal standard to quantify the analyses. The CaO content of NIST 610 used in the calculation is 11.45 wt.%. The data were reduced using the *GLITTER* 4.0 program.

Oxygen isotope compositions of olivine were analyzed in situ using CAMECA IMS-1280 ion microprobe at IGGCAS. Sample grains were prepared in epoxy adjacent to grains of a San Carlos olivine intralaboratory standard and then polished to a flat, smooth surface. The Cs^+^ primary beam was accelerated at 10 kV with an intensity of ca. 2 nA. The spots size was about 20 μm in diameter (10 μm beam diameter plus 10 μm raster). An electron gun was used to compensate for sample charging during analysis. Secondary ions were extracted with a ~10 kV potential. Oxygen isotopes were measured in multi-collector mode with two off-axis Faraday cups with each analysis consisting of 16 cycles × 4 s counting time. The instrumental mass fractionation factor is corrected using San Carlos olivine standard with δ^18^O_VSMOW_ = 5.25‰ (ref. 33). Point-to-point uncertainty for δ^18^O was typically better than 0.4‰ (2 SD).

## Author Contributions

C.Z.L., F.Y.W. and W.Q.J. collected the samples. C.Z.L., W.Q.J. and Q.L.L. conducted the analyses. C.Z.L., F.Y.W., S.L.C. and W.D.S. contributed to discussions, interpretation of results and manuscript writing.

## Supplementary Material

Supplementary Information Supplementary Text

Supplementary Information ST1

Supplementary Information ST2

Supplementary Information ST3

## Figures and Tables

**Figure 1:**
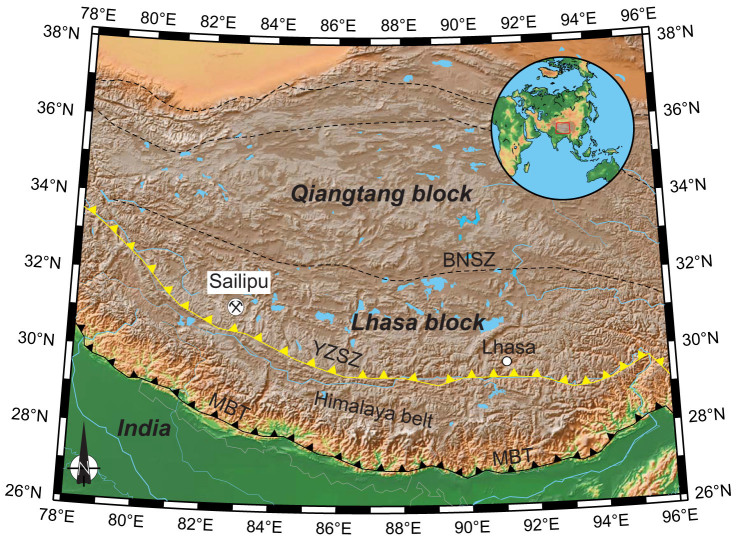
Sketch map of the Tibetan Plateau. Yellow line marks the Yarlung-Zangbo Suture Zone (YZSZ), representing the plate boundary along which northward subduction of the Neo-Tethyan oceanic lithosphere took place before the India-Asia collision. The Sailipu mantle xenoliths crop out ~50 km north of the Yarlung-Zangbo Suture Zone. BNSZ: Banggong-Nujiang Suture Zone. MBT: Main boundary thrust. (This figure is generated by Chuan-Zhou Liu, using Generic Mapping Tool (GMT) package developed by Wessel and Smith^34^).
