# Supplementary material for: A ‘hidden’ 18O-enriched reservoir in the sub-arc mantle
Source: Sci Rep. 2014 Feb 28;4:4232. doi: 10.1038/srep04232 (PMC3937801; doi:10.1038/srep04232)
Supplement: Supplementary Information — Supplementary Text [file srep04232-s1.doc]

Supplementary Information

The ‘hidden’ ^18^O-enriched reservoir in the sub-arc mantle

Chuan-Zhou Liu^1,*^, Fu-Yuan Wu^1^, Sun-Ling Chung^2^, Qiu-Li Li^1^, Wei-Dong Sun^3^, Wei-Qiang Ji^1^

1. *State Key Laboratory of Lithospheric Evolution, Institute of Geology and Geophysics, Chinese Academy of Sciences, Beijing 100029, China*

*2. Department of Geosciences, National Taiwan University, Taipei 10617*

*3. State Key Laboratory of Isotope Geochemistry, Guangzhou Institute of Geochemistry, Chinese Academy of Sciences, Guangzhou 510640, China*

**This PDF file includes:**

Supplementary Text;

Supplementary Table S1, S2 and S3.

**Supplementary Text: petrographic description**

Ultrapotassic lavas erupted in Sailipu, southwestern (SW) Tibet, contain both mantle and crustal xenoliths. Mantle peridotite xenoliths are small in size, with diameters commonly less than 2 cm (Liu et al., 2011). The Sailipu mantle xenoliths display porphyroclastic to equigranluar textures (Fig. S1). The SLP105 is the most fresh sample among all studied Sailipu mantle xenoliths (Fig. S1b). They are mainly composed of olivine and orthopyroxene, whereas clinopyroxene is only present in five samples. Olivine commonly shows kink bands and triple junction is well developed in most samples. Phlogopite is a minor phase present in all samples but SLP105, which is commonly interstitial among other silicate minerals (i.e., olivine, clinopyroxene and orthopyroxene). It has been previously reported that the modal content of phlogopite could be as high as 5% in some Sailipu mantle (Liu et al., 2011). Occasionally, phlogopite shows a reaction texture with spinel, in which spinel is replaced by phlogopite (Fig. S1f; Liu et al., 2011). Spinel has been only observed in three samples. Spinel in SLP105 occurs as a inclusion in orhtopyroxene (Fig. S1b), whereas it is interstitial among silicates in both SLP127 and SLP154. Some mantle xenoliths show reaction rims with the host lavas, in which orthopyroxene is formed in the boundary.

**Reference**

Liu, C. Z., Wu, F. Y., Chung, S. L. & Zhao, Z. D. Fragments of hot and metasomatized mantle lithosphere in Middle Miocene ultrapotassic lavas, southern Tibet. *Geology* **39**, 923-926 (2011).


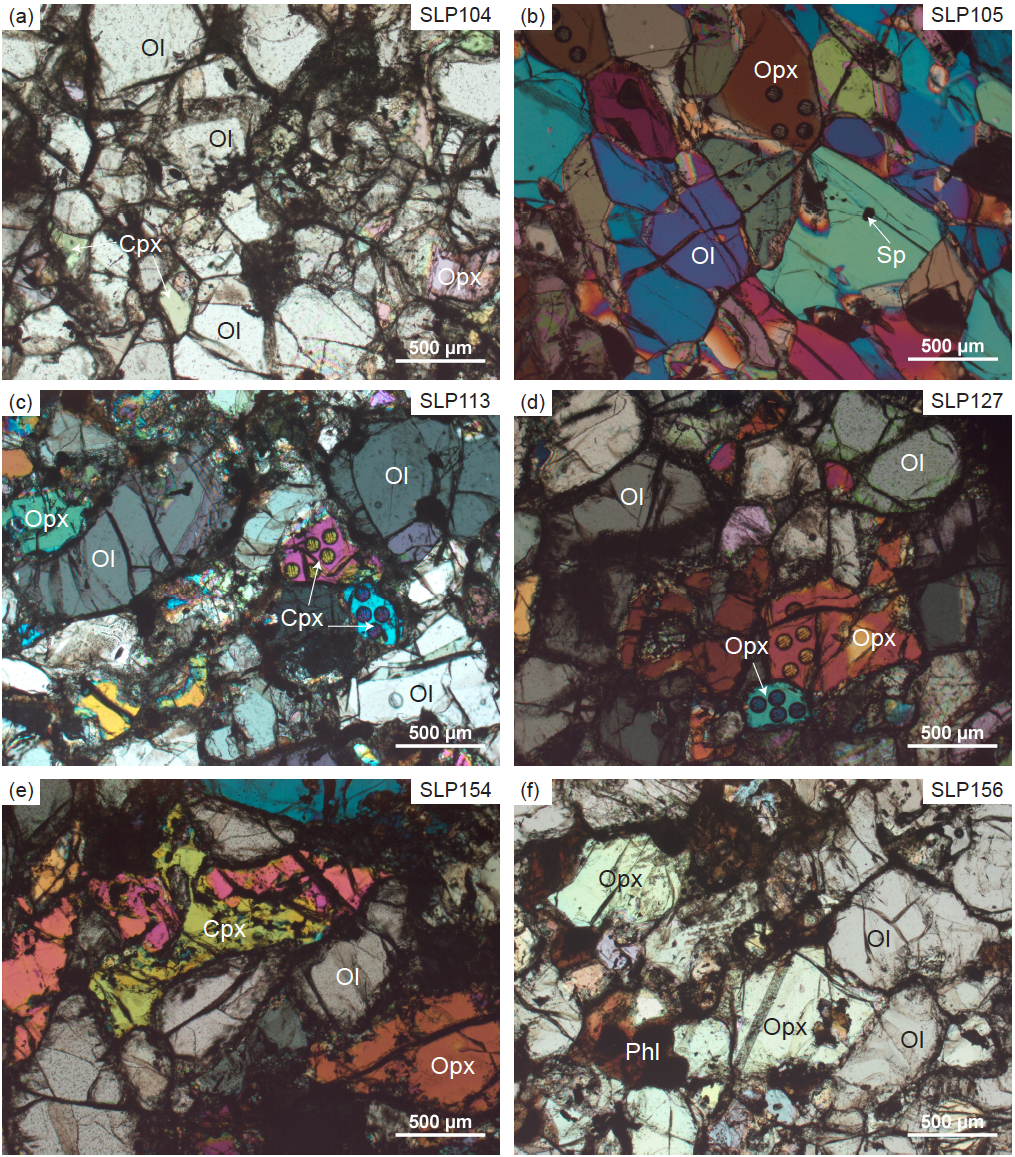


***Fig. S1: Microtextural images of six studied Sailipu mantle xenoliths.*** *Ol: olivine; Opx: orthopyroxene; Cpx: clinopyroxene; Phl: phlogopite; Sp: spinel.*
